# Assessing the Distribution of Potentially Toxic Elements in Bryophytes in Relation to Surface Soil Contamination in the Veles Region, North Macedonia

**DOI:** 10.3390/plants14050783

**Published:** 2025-03-04

**Authors:** Trajče Stafilov, Katerina Bačeva Andonovska, Robert Šajn, Marija Jeftimova

**Affiliations:** 1 Institute of Chemistry, Faculty of Natural Sciences and Mathematics, Ss Cyril and Methodius University in Skopje, Arhimedova 5, 1000 Skopje, North Macedonia; trajcest@pmf.ukim.mk (T.S.); marija_jeftimova@yahoo.com (M.J.); 2 Research Center for Environment and Materials, Macedonian Academy of Sciences and Arts, 1000 Skopje, North Macedonia; 3 Geological Survey of Slovenia, Dimičeva 14, 1000 Ljubljana, Slovenia; robert.sajn@geo-zs.si

**Keywords:** moss biomonitoring, air pollution, surface soils, potentially toxic elements, Veles region, North Macedonia

## Abstract

This study explores the relationship between bryophyte (mosses) diversity and environmental factors in the Veles region, North Macedonia, focusing on the spatial distribution of chemical elements in the moss and surface soil samples collected from the same locations. Eighteen moss samples were analyzed alongside surface soils. Advanced spectrometric techniques were used to identify potentially toxic elements (PTEs) and their links to anthropogenic and natural sources. While metal measurements are widely reported in the literature, the novelty of this study lies in its integrative approach, combining moss biodiversity analysis with a direct comparison of element concentrations in both moss and soil. The results show significant patterns of deposition of PTEs and highlight the long-term impact of industrial activities on biodiversity and air pollution. These findings provide valuable insights into conservation strategies and environmental management in the midst of ongoing ecological change. Five groups of elements were separated using factor analysis: G1 (Al, Cr, Cu, Fe, Li, Mg, Mn, Ni and V); G2 (Ba and Na); G3 (K, P and Mo), G4 (Pb and Zn), and G5 (Ag, As and Cd), of which two groups (G1 and G2) were found to be typical geochemical associations, while G4 and G5 are anthropogenic associations due to the emission of dust from contaminated soils and the slag heap of the Pb-Zn smelting plant. Group 3 represents a mixed geochemical and anthropogenic association. It was found that Pb, Zn, Cd, and As could indeed be detected in the moss in the study area, underlining its ability to detect pollutants in the air. A comparative analysis of moss and soil samples revealed significant differences in element concentrations, with most elements being more concentrated in soil. These results underline the role of moss as a bioindicator of atmospheric deposition, detecting pollution trends rather than direct soil contamination.

## 1. Introduction

Biomonitoring is a technique in which plant species are used to detect or predict environmental changes. Mosses are often used for this purpose, as they obtain most of their nutrients directly from dry and wet depositions, which are particularly favorable for the accumulation of potentially toxic elements (PTEs) [1,2,3,4,5,6,7,8,9,10,11,12,13,14,15,16,17]. PTEs constitute a significant proportion of environmental pollutants, with lead (Pb), cadmium (Cd), zinc (Zn), mercury (Hg), arsenic (As), and chromium (Cr) among the most critical [18]. Bryophytes, especially mosses, have the unique ability to take up and accumulate one or more elements in concentrations that are toxic to other plant species. These elements are mainly taken up from the atmosphere, although they can also be absorbed from the soil. Older moss tissue often contains higher concentrations of these elements than younger tissue [19,20].

Mosses are highly effective indicators of air pollution due to their unique physiology and ecological role. Unlike vascular plants, mosses have no roots and obtain their nutrients directly from the air rather than the soil, making them excellent monitors of air pollution. This characteristic has made them a key component of the global biomonitoring of air pollution, especially for the assessment of PTEs. The European moss study on air pollution, within the International ICP Vegetation Program, collects and analyzes moss samples across Europe every five years [21,22,23]. North Macedonia has participated in this program since 2002 [24], collecting moss samples in 2005, 2010, 2015, and 2020 [25,26,27,28,29]. The results consistently indicate increased air pollution with PTEs, especially in areas affected by mining and metallurgical activities.

This study builds on previous studies of air pollution in the Veles region, where industrial activities, particularly the abundant Pb-Zn smelter, have left a lasting legacy of pollution. The region’s soil and moss samples were compared to assess air pollution, as moss biomonitoring reflects air pollution, while soil samples often show both airborne and geochemical sources of PTEs. Studies on the environmental impact of the Pb-Zn smelter in Veles have revealed extensive soil pollution with various PTEs, including As, Cd, Cu, Hg, In, Pb, Sb, and Zn [28]. These pollutants affect air quality through particulate emissions and have also been detected in locally produced food, posing a risk to human health and ecosystems [29,30,31,32].

The dominance of anthropogenic sources in the contamination is reflected in the elevated concentrations of PTEs in dust samples from urban Veles compared to nearby rural and mountainous areas. The proximity to the smelter and its slag landfill, which is rich in toxic elements, has contributed significantly to the persistent pollution, even after the closure of the smelter. Household dust also shows that contaminated soils continue to act as a secondary source of air pollutants, keeping indoor exposure at high levels. The persistence of toxic elements such as As, Cd, and In at alarming levels poses a significant threat to human health and the local ecosystem. Factor analysis reveals the anthropogenic association of elements such as Cd, Pb, and Zn with smelting activities, differentiating them from geogenic contributions such as Co, Cr, and Ni. This study provides a critical understanding of historical and current pollution in Veles and emphasizes the need for mitigation and monitoring measures to address environmental and public health challenges in the region [29].

Previous studies have shown that the city of Veles is significantly contaminated by potentially toxic elements (PTEs) due to emissions from a lead and zinc smelter that was in operation until 2002 [29,30,31,32]. High concentrations of PTEs, including Cd, Pb, Zn, As, Co, Ag, In, Sb, and Se, were detected in attic and household dust samples, reflecting the long-term deposition of these elements in urban areas [29]. A comparison of PTE concentrations in the different zones revealed striking differences. The urban zone (city of Veles) showed the highest contamination levels. Thus, the Cd content in attic dust of the houses in the urban zone reached 240 mg/kg, compared to 56 mg/kg in the zone of the nearby village of Bašino Selo and 4.6 mg/kg in the mountain zone (Klepa Mountains). A similar correlation was also found for the other PTEs. This study builds on previous research on air pollution in the Veles region, particularly in relation to the Pb-Zn smelter. While previous studies focused primarily on soil pollution and the presence of PTEs such as As, Cd, Cu, Hg, Pb, Sb, and Zn in soils and food, this study introduces a new approach by directly comparing moss and soil samples from the same sites. Mosses are well-established bioindicators of air pollution, and our research provides new insights into how atmospheric deposition of PTEs differs from soil contamination, with a focus on airborne pollutants. By conducting a factor analysis, we distinguish between anthropogenic sources (such as emissions from the Pb-Zn smelter) and geogenic sources (such as Co, Cr, and Ni), which provides a deeper understanding of pollution dynamics in the region. These results provide important new data on the ongoing environmental problems in Veles and emphasize the need to continue to reduce and monitor pollution to protect public health and ecosystems in the region.

The motivation for this study is to use moss biomonitoring to map the spatial distribution of PTEs and identify the main sources of pollution in Veles. Moss samples were collected from 18 sites, and after microwave digestion, 21 elements were analyzed using inductively coupled plasma–atomic emission spectrometry (ICP-AES). Factor analysis was applied to determine correlations between the analyzed elements, taking into account both geochemical similarities and anthropogenic influences. The results were then used to create distribution maps for the identified factor groups, focusing on elements of lithological and anthropogenic origin. The comparison of moss and soil samples taken from the same locations strengthens the ability to distinguish air pollution from geochemical and anthropogenic sources. This integrated approach contributes to a deeper understanding of pollution and its spatial patterns in the region.

## 2. Results and Discussion

The basic statistics for the 21 elements (Ag, Al, As, Ba, Ca, Cd, Cr, Cu, Fe, K, Li, Mg, Mn, Mo, Na, Ni, P, Pb, Sr, V, and Zn) in the moss samples from the studied region (*n* = 18) are presented in Table 1. The concentrations of Al, Ca, Fe, K, and Mg are expressed as percentages, Ag and Mo as µg/kg, and the remaining elements as mg/kg.

The median values for the macroelements are as follows: Ca (0.48%), K (0.26%), Al (0.16%), Fe (0.12%), Mg (0.12%), and P (0.057%). The trace elements are ordered by their median concentrations: Na (90 mg/kg), Mn (60 mg/kg), Zn (27 mg/kg), Ba (21 mg/kg), Sr (16 mg/kg), Cu (4.9 mg/kg), Ni (4.8 mg/kg), Cr (2.7 mg/kg), V (2.2 mg/kg), Pb (2.1 mg/kg), Li (1.3 mg/kg), As (0.66 mg/kg), Cd (0.30 mg/kg), Mo (0.10 mg/kg), and Ag (0.073 mg/kg).

The descriptive statistics of the contents of the same analyzed elements in soil samples collected from the same locations are given in Table 2, in which the values were calculated for a total of 18 topsoil samples. The values are given in mg/kg. The median values for the analyzed elements reveal the following order for macroelements (given in percent): Al (2.1%), Fe (2.2%), K (1.5%), Ca (1.1%), Na (1.1%), and Mg (0.46%). The trace elements have the following order according to the value of the median content: Mn (440 mg/kg), Ba (380 mg/kg), P (300 mg/kg), Zn (96 mg/kg), Sr (74 mg/kg), Cr (100 mg/kg), Cu (15 mg/kg), Ni (39 mg/kg), V (50 mg/kg), Li (12 mg/kg), Pb (30 mg/kg), B (1.2 mg/kg), Ag (0.75 mg/kg). The distribution reflects the natural variability and the geochemical properties of the elements in the samples.

Table 3 shows the comparison of the median, minimum, and maximum values for the contents of the analyzed elements in the moss from the Veles region with the values for the entire area of North Macedonia, which were collected in 2015 [27]. It was found that the median values for the content of almost all analyzed elements in the moss samples from the Veles region are very similar to the values for North Macedonia. The only differences are that the content of As, Cu, and Ni in some specific areas of the Veles region is higher than in the moss samples from the entire territory of Macedonia due to the lithological and anthropogenic influences, and the content of some elements is lower due to the presence of polluted areas in the country due to mining and smelting activities.

The degree of correlation between the content of the chemical elements in the moss samples was assessed using the Pearson correlation coefficient (*r*). A qualitative interpretation was applied in which absolute *r* values between 0.45 and 0.7 were considered to indicate a moderate correlation, corresponding to a coefficient of determination between 25% and 50%. Correlation coefficients greater than 0.70 were considered to indicate a strong association, reflecting a very similar distribution of the elements. The concentrations of the individual elements were correlated with those of the other elements [33]. All correlation coefficients for the elements are shown in the correlation matrix in Table 4. The correlation coefficients of the contents of the analyzed elements show that there are strong correlations between several elements, e.g., between the following: Al and Ba; Fe, Li, Mn and V; Ba and Fe; Ni and V; Cr and Ni and V; Cu and Mg; Fe and Li; Mn and V; Li and Mn and V; Mg and P; Mn and V; Ni and Pb; and Sr and V.

Based on these results, a factor analysis was carried out, and five associations (groups) of elements were identified: Group 1 (Al, Cr, Cu, Fe, Li, Mg, Mn, Ni and V); G2 (Ba and Na); G3 (K, P and Mo), G4 (Pb and Zn), and G5 (Ag, As and Cd), interpreted as factors G1–G5. Calcium was excluded from further analysis as it tends to form its own factor that is not related to other elements.

**Figure 1:**
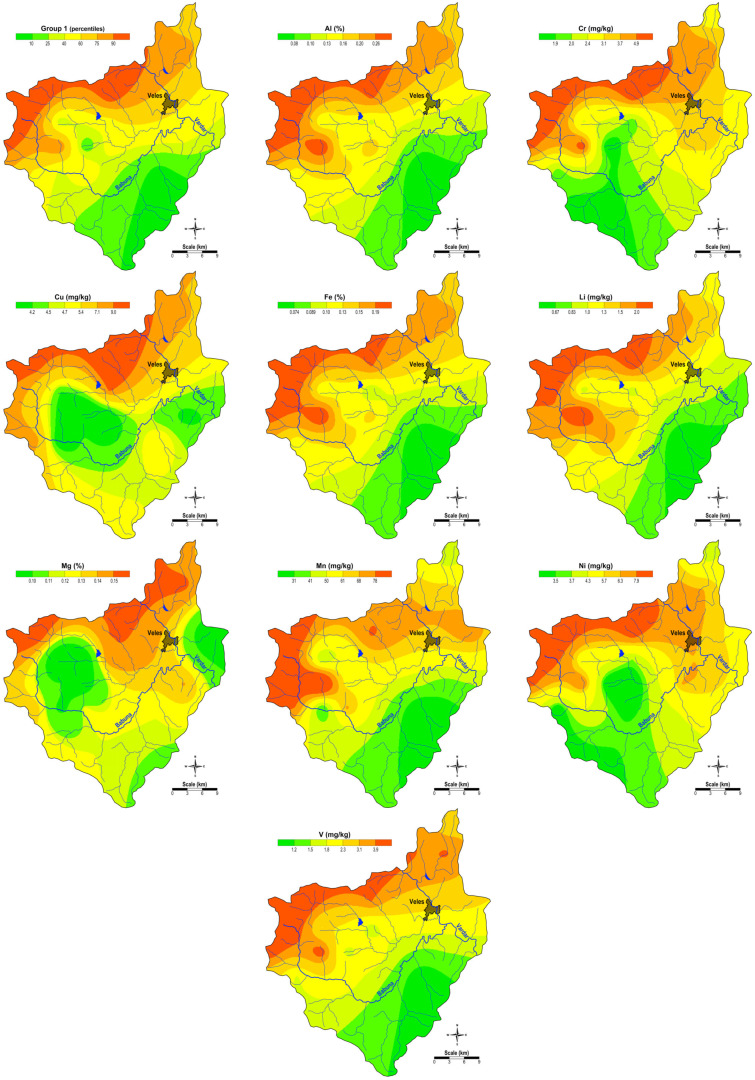
Spatial distribution of factor values for G1 and the content of the Group 1 elements (Al, Cr, Cu, Fe, Li, Mg, Mn, Ni, and V).

Group 1 (Al, Cr, Cu, Fe, Li, Mg, Mn, Ni, and V) represents a lithogenic combination of elements. The spatial distribution of the factor scores of G1 is shown in Figure 1. The presence of these elements in this typical lithogenic association is linked to the geological composition of the soil in the region. This is further supported by the fact that, with the exception of Al, all these elements (Cr, Cu, Fe, Li, Mg, Mn, Ni, and V) are also found in the Factor 1 association from the factor analysis of the elemental content in the soils from the same area [34]. From the distribution maps (Figure 1), it can be seen that the high contents of the elements from G1 occur in the northern and northwestern parts of the study area, where there are Precambrian gneisses and ancient granites, Precambrian and Palaeozoic schists, and, in the northern part, Tertiary sediments (where their content in the soil is also increased) [34]. In addition, there is a certain increase in the Cr and Ni content in moss samples from the surroundings of the town of Veles, which could also be due to the influence of air pollution with dust from the work of the ferronickel smelter in Kavadarci [26,27,35].

**Figure 2:**
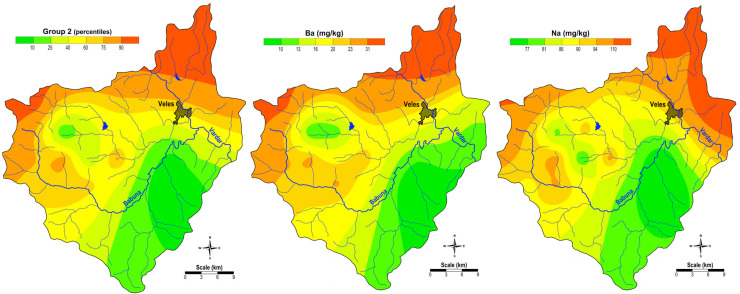
Spatial distribution of factor values for G2 and the content of the Group 2 elements (Ba and Na).

The map of the spatial distribution of the factor scores of Group 2 and the spatial distribution of the elements of this factor (Ba and Na) are shown in Figure 2. This association is also a typical lithogenic association related to the geological composition of this particular area. The distribution maps show that these elements are present in higher contents in the northeast, where Tertiary sediments predominate, and in the northeast of the study area, where Precambrian gneisses and old granites predominate.

**Figure 3:**
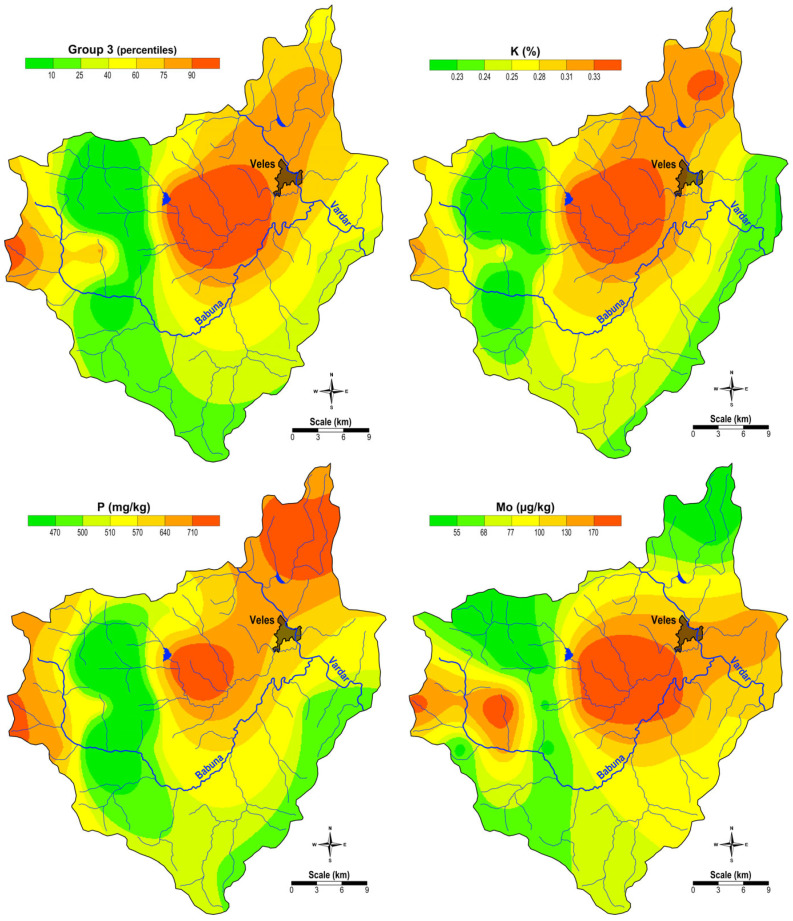
Spatial distribution of the factor values for G3 and the content of the elements of Group 3 (K, P, and Mo).

Group 3 includes K, P, and Mo, with elevated concentrations of these elements found in moss samples from the central part of the region (Figure 3), where agricultural activities are prevalent. This suggests that the increased content of K and P in the soils of this area is likely due to the use of artificial fertilizers containing these elements.

**Figure 4:**
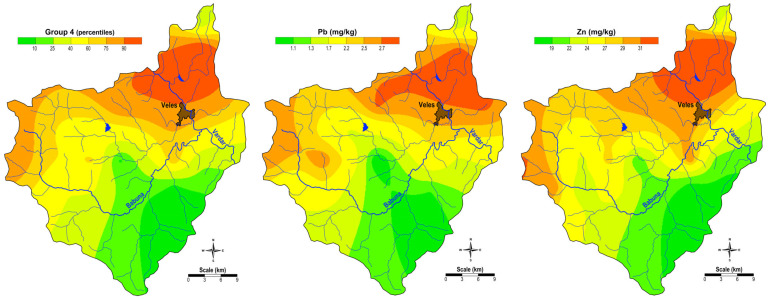
Spatial distribution of factor values for G4 and the content of Group 4 elements (Pb and Zn).

The spatial distribution of the factor scores for Group 4 (Pb and Zn), shown in Figure 4, indicates an anthropogenic association, with the highest concentrations in moss samples from the areas surrounding the town of Veles. This pattern reflects the influence of the former Pb-Zn smelter “Zletovo” on the local environment. This is also confirmed by the fact that both elements are also represented in the Factor 2 association from the factor analysis (Pb and Zn) of the content of the elements in the soils of the same area [34]. Although the smelter for Pb and Zn in this town has been closed since 2003, the high soil pollution and dust emissions from the slag heap due to the work of the smelter still result in air pollution from dust enriched with these potentially toxic elements [26,28].

**Figure 5:**
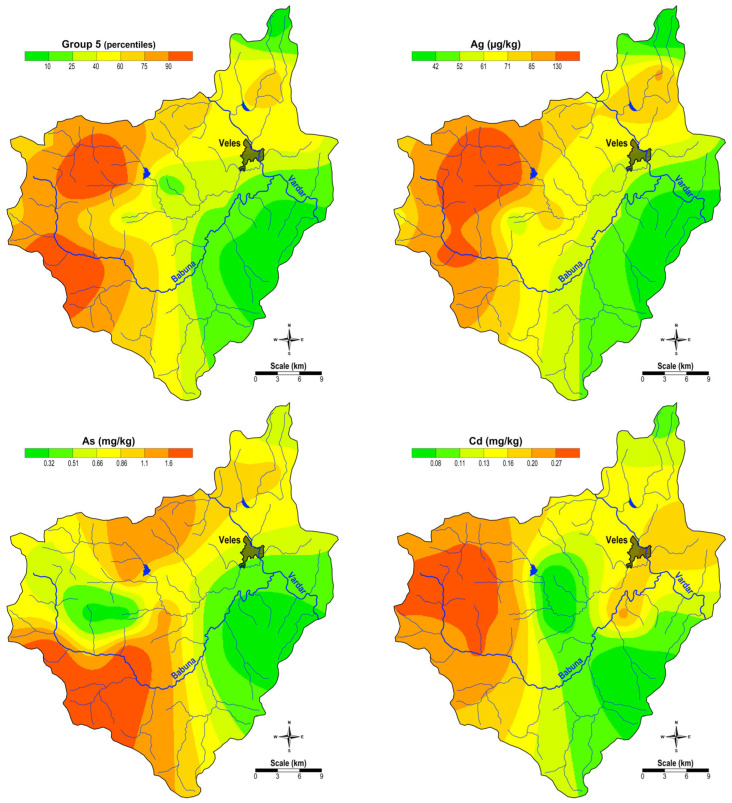
Spatial distribution of the factor values for G5 and the content of Group 5 elements (Ag, As, and Cd).

The spatial distribution of the factor scores of Group 5 (Ag, As, and Cd) and the content of Ag, As, and Cd are shown in Figure 5. It can be seen that an increased content of these elements is found in the moss samples from the western part of the study area, where Precambrian gneisses and old granites predominate, and in the area around the town of Veles, which is due to the emission of polluted soils and Pb-Zn slag from smelting, which also contain these elements. The significant deposition patterns relate to the higher concentrations of specific elements such as Pb, Zn, Cd, and As found in moss samples near the industrial site, indicating airborne deposition. These patterns indicate a strong influence of industrial emissions on air pollution, with moss serving as an effective bioindicator of such depositions.

Moss samples in the Veles region showed elevated concentrations of potentially toxic elements (PTEs) such as lead, zinc, and cadmium, especially in urban areas near the former smelter site. Factor analysis showed clear associations of elements, distinguishing between geogenic and anthropogenic sources, with Pb and Zn strongly associated with industrial emissions (Figure 4).

The high concentrations of PTEs detected in moss samples indicate potential risks to moss diversity and overall ecosystem health. Mosses play a crucial role as bioindicators due to their ability to accumulate PTEs. However, their susceptibility to pollution highlights how vulnerable they are and raises concerns about the wider ecological impacts of pollution in the region, which, in turn, negatively impacts public health.

The *t*-test could play an important role in identifying statistically significant differences in the concentrations of individual elements between soil and moss samples. Quantifying these differences provides essential insights into how pollutants are distributed and retained in different environmental media. This analysis highlights the complementary role of soil and moss, with soil serving as an indicator of long-term deposition and moss reflecting current air pollution. Understanding these dynamics is crucial for assessing the extent and sources of pollution and for defining effective environmental management and remediation strategies.

The results of the *t*-test presented in Table 5 show significant differences in the concentrations of most elements, including Ag, Al, As, Cr, Fe, K, Ni, Pb, and Zn, between the soil and moss samples. These results suggest that moss does not directly reflect soil concentrations but picks up additional sources of contamination, probably from the air. This difference underlines the importance of using moss as a bioindicator for air pollution monitoring.

Among the elements with non-significant differences, barium is significant at *p* = 0.008, indicating a notable but less pronounced difference compared to the other elements. Calcium is significant at *p* = 0.026, indicating a notable difference. In contrast, molybdenum shows no significant difference (*p* = 0.073), which could indicate similar concentrations in moss and the soil. No data are available for cobalt (NaN). One or both variables do not contain enough observations (indicates values below the LOD), so no conclusion can be drawn.

The descriptive statistics show a general trend where most elements have higher concentrations in soil than in moss, which is consistent with the assumption that soil serves as the primary reservoir for these elements. The concentrations in moss are significantly lower, reflecting its role as a biological indicator of atmospheric deposition and not directly corresponding to soil. Levels in moss are significantly lower, reflecting its role as a biological indicator of atmospheric deposition and not directly corresponding to soil.

Notable outliers include phosphorus, whose concentrations are much higher than in soil, indicating a possible external source or bioaccumulation in plant tissue. Calcium shows extreme variability in soil concentrations, while moss concentrations are more consistent.

The interpretation of the data shows that the moss effectively absorbs elements from the air, especially Pb, Zn, Cd, and Cr, which are common pollutants from industrial and traffic sources. The large differences in the *t*-test statistics (e.g., Pb: *t* = 11.59, Zn: *t* = 11.96) further support this.

Paired samples *t*-test results are presented with detailed components, including the *t*-value, degrees of freedom (*df*), and *p*-value. Higher *t*-values indicate greater differences between the two groups, and a *p*-value of less than 0.05 suggests statistically significant differences. The results show that most elements, including Ag, Al, As, Cd, Fe, and Zn, have *p*-values below 0.001, indicating significant differences between the soil and moss groups. Phosphorus, for example, shows a large negative mean difference of −15.61 mg/kg, suggesting a significant reduction in moss compared to soil. Potassium (K), sodium (Na), and aluminum (Al) also show significant differences.

Arsenic has a *t*-value of 49.75 and a *p*-value of less than 0.001, indicating a significant difference between the two groups. The mean arsenic concentration in the soil is 10 mg/kg and shows minimal variability, while the moss samples have a lower mean value of 0.88 mg/kg with greater variability. This difference indicates limited arsenic accumulation in the moss, possibly due to limited uptake pathways or distribution dynamics in the environment.

For copper, a *t*-value of 4.14 and a *p*-value of less than 0.001 confirm a statistically significant difference. The soil samples contain an average of 12.25 mg/kg, in contrast to the moss samples, whose average value is 6.2 mg/kg. This pattern emphasizes the relatively lower ability of moss to absorb copper from its environment.

Nickel has a *t*-value of 3.36 and a *p*-value of 0.004, indicating a significant difference between soil and moss samples. The mean nickel concentration is 54.9 mg/kg in soil, which is significantly higher than the 5.70 mg/kg found in moss. This discrepancy probably reflects the limited bioavailability of nickel in moss or its inability to effectively store the element.

Cadmium shows a remarkable difference between the two groups, with a *t*-value of 18.04 and a *p*-value below 0.001. The soil samples contain an average of 2.25 mg/kg, while the moss samples have an average of only 0.18 mg/kg. This result indicates that cadmium is largely unavailable to the moss or is actively excluded from its tissue.

The lead analysis gives a *t*-value of 11.59 and a *p*-value of less than 0.001, confirming a significant difference. The mean lead concentration in soil is 26.45 mg/kg, while in moss, it is 2.10 mg/kg. This indicates that the lead in the moss comes primarily from the air and not through uptake from the soil.

Zinc shows a *t*-value of 11.96 and a *p*-value of less than 0.001, indicating a significant difference between the groups. The soil samples show an average zinc concentration of 86.01 mg/kg, while the moss samples show an average of 26.67 mg/kg. This distribution indicates that moss primarily reflects the bioavailable or atmospheric fraction of zinc and not the total content of the soil.

In summary, the results confirm significant differences in elemental composition between moss and soil, with moss providing a clear snapshot of bioavailable and airborne pollutants. These results emphasize the importance of moss as a valuable tool for environmental assessments and contaminant studies, providing insights into the presence of bioavailable contaminants in the ecosystem.

**Figure 6:**
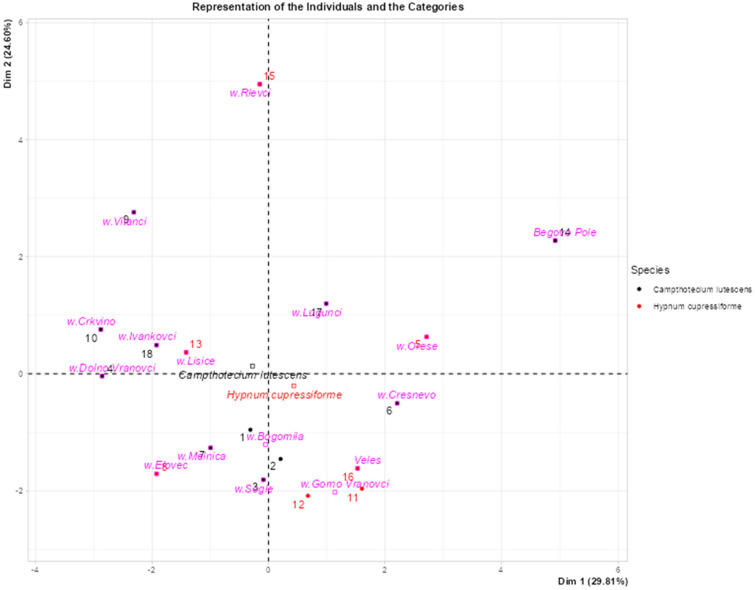
Principal component analysis (PCA) biplot that visualizes the distribution of individuals and categories.

The diagram in Figure 6 represents the principal component analysis (PCA), where the concentrations of potentially toxic elements were used as active variables and transformed into principal components (Dim 1 and Dim 2), which capture the highest variance in the data set. Moss species and sampling locations were included as supplementary variables projected onto the biplot based on their relationships with PTE concentrations without affecting the PCA calculation. This method facilitates the visualization of patterns in PTE accumulation and highlights ecological or geographical relationships. The PCA biplot visualizes the distribution of individuals (sites) and categories (moss species) by projecting them onto a two-dimensional space defined by the first two principal components (Dim 1 and Dim 2).

The distribution of individuals and categories on the biplot reflects their similarities or differences based on PTE concentrations. Individuals that are close to each other have similar PTE profiles, while those that are farther apart differ significantly. The projection of moss species and locations shows how these additional variables relate to the principal components and highlights possible ecological or geographical trends in PTE accumulation.

Different colors are used to distinguish species, such as *Camptothecium lutescens* in black and *Hypnum cupressiforme* in red, to facilitate differentiation at the species level. Clusters of data points indicate similarities within species or sampling locations, while more dispersed points indicate greater variability.

Categories, such as sampling locations (e.g., w. Vitanci and w. Oreše), are shown in pink and red. Their proximity to individuals on the diagram reflects correlations, with points closer together representing similarities, while those further apart indicate differences in the measured variables. The diagram is divided into four quadrants by dashed lines at the origin. The distribution across these quadrants shows possible patterns, such as different groupings of species or sampling points.

Key observations include the proximity of points, the formation of clusters, and the identification of outliers. For example, sampling points such as MK-494 (near Bogomila) and MK-623 (near Veles) are close to each other, indicating similar profiles, while sampling points such as MK-619 (near Rlevci) and MK-539 (near Vitanci) are further apart, indicating different characteristics. Clusters, such as the grouping of MK-539 (w. Vitanci) and MK-540 (w. Crkvino), indicate common features, while isolated sites, such as w. Rlevci (MK-619) or MK-616 (from Begovo Pole), could indicate unique features or potential outliers.

**Figure 7:**
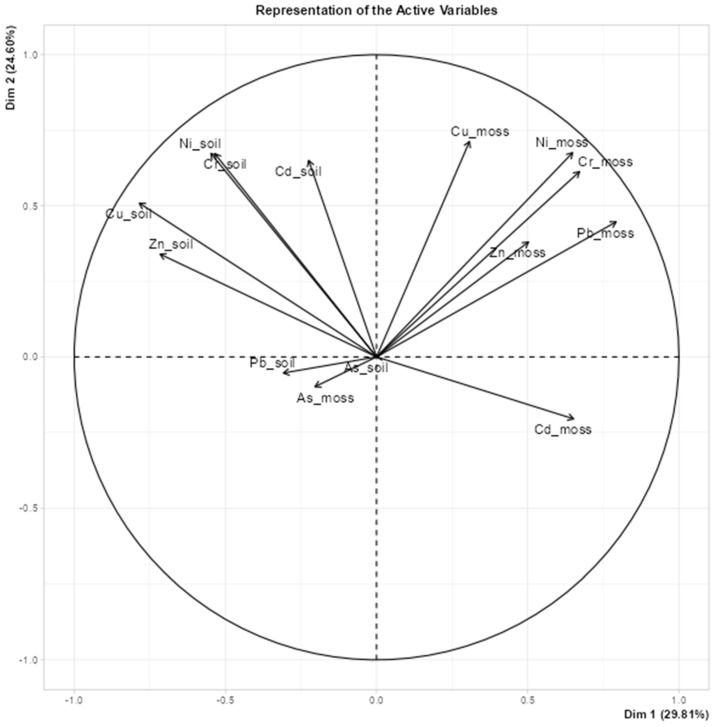
Representation of the PTE variables.

Figure 7 shows a diagram created using principal component analysis (PCA) to visualize the relationships between active variables, in particular, the concentrations of potentially toxic elements (PTEs) such as Ni, Cd, Cu, Zn, Pb, and As in soil and moss. This diagram shows how these variables contribute to the first two principal components (Dim 1 and Dim 2). Dim 1 explains 23.8% of the variance and Dim 2 20.4%, which together account for 44.2% of the total variance. This indicates that the two dimensions capture a substantial part of the structure of the data.

In the plot diagram, each vector represents a variable (e.g., Ni_soil or Cd_moss), with its direction and length indicating its relationship to the principal components. Variables aligned with Dim 1, on the horizontal axis, explain a large proportion of the variance for that component, while those aligned with Dim 2 on the vertical axis make a significant contribution. For example, Cd_soil and Zn_soil might be primarily aligned with Dim 1, while Cu_moss and Cr_moss contribute more to Dim 2. The plot also helps to interpret the relationships between PTE concentrations in soil and moss. For example, the arrows for the same PTE in soil and moss, such as Ni_soil and Ni_moss, point in the same direction, suggesting similar environmental influences.

The angles between the vectors indicate the type of correlations: small angles indicate positive correlations, angles of about 90 degrees indicate no correlation, and angles close to 180 degrees indicate negative correlations. Therefore, Ni_moss and Cu_moss are positively correlated, while As_moss and Cd_soil are probably negatively correlated. This correlation circle diagram also identifies clusters of variables that behave similarly, such as Ni_moss, Cu_moss, and Cd_moss, and distinguishes variables, such as As_moss, that behave differently. These patterns could indicate differences in the properties or origin of the soil and moss variables.

**Figure 8:**
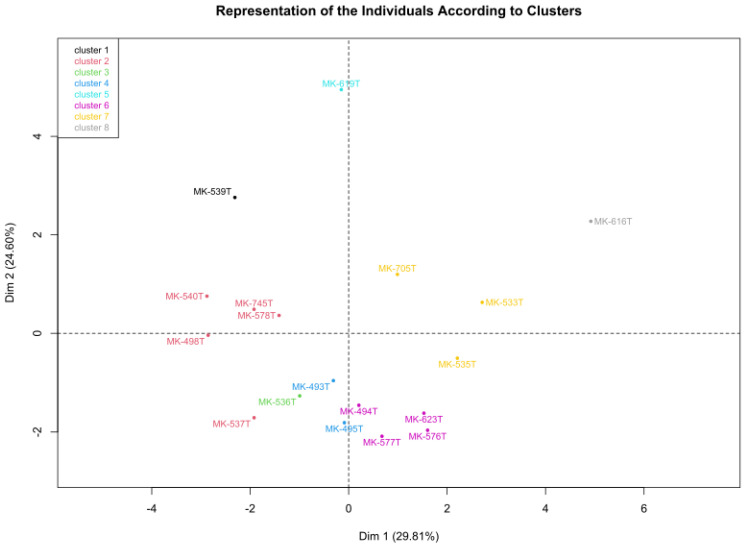
Represents the clustering of the individual samples on the first two principal components.

The plot visualization of the clustering of the individual sample on the first two principal components (Dim 1 and Dim 2) from a PCA is presented in Figure 8. Each sample, such as “MK-539” Cluster 1 or “MK-619 Cluster 5”, is assigned to a cluster, with the colors representing different groups.

The clusters in the diagram are generally well separated and show different groupings based on the principal components. For example, Cluster 1 contains “MK-539”, which is positioned in isolation from the other clusters, while Cluster 5 occupies the upper right part of the plot “MK-619”. Outliers such as “MK-539” Cluster 1 and “MK-616” Cluster 8 may represent unique conditions or local anomalies that differ significantly from the general trends in the data set. The clusters represent similarities or differences in chemical signatures, such as variations in element concentrations. For example, soil and moss samples collected at the same location could belong to the same cluster if they have similar pollution profiles. However, differences between clusters indicate different local conditions or differences in the behavior of pollutants in soil compared to moss.

## 3. Conclusions

The moss biomonitoring technique has demonstrated its effectiveness as a method for assessing the atmospheric deposition of potentially toxic elements (PTEs) in the Veles region, North Macedonia. By analyzing 21 chemical elements (Ag, Al, As, Ba, Ca, Cd, Cr, Cu, Fe, K, Li, Mg, Mn, Mo, Na, Ni, P, Pb, Sr, V, and Zn), valuable insights into pollution sources and environmental conditions were obtained. The factor analysis identified five different element associations that provide information on the geochemical and anthropogenic contributions to pollution. Groups G1 (Al, Cr, Cu, Fe, Li, Mg, Mn, Ni, and V) and G2 (Ba and Na) represent natural geochemical associations and reflect the soil composition and geological background. In contrast, Group G4 (Pb and Zn) indicates anthropogenic sources, mainly emissions from the slag heap of the Pb-Zn smelter and contaminated soils in the vicinity of the smelter in the town of Veles. This group underlines the long-term environmental legacy of industrial activities in the area. Mixed associations were also observed: Group G3 (K, P, and Mo) was associated with agricultural activities, while Group G5 (Ag, As, and Cd) was associated with soil pollution and residues from the former smelter. The presence of elements such as Pb, Zn, Cd, and As in moss samples in the vicinity of the smelter underlines the role of airborne deposition and secondary emissions from contaminated soils.

Comparative analysis of moss and soil samples revealed significant differences in element concentrations, with most elements being more concentrated in the soil. These results underline the role of moss as a bioindicator of atmospheric deposition, detecting pollution trends rather than direct soil contamination. In particular, elements such as Pb, Zn, Cd, and As could be detected in moss, highlighting its ability to detect air pollutants. Lead and zinc, in particular, showed the greatest differences between soil and moss samples, with the moss reflecting airborne deposition from the air by smelter emissions and the slag heap. These results highlight the utility of moss in detecting atmospheric pollution, while soil reflects long-term accumulation and local contamination.

The results of this study emphasize the urgent need for sustained environmental monitoring, effective remediation strategies, and targeted protective measures against pollution and its consequences. The protection of moss biodiversity and the restoration of degraded habitats require integrated approaches that prioritize pollution reduction and the development of sustainable strategies.

Moss biomonitoring provides an efficient, cost-effective, and non-invasive method for assessing atmospheric pollution. While it has its limitations, particularly in terms of detecting soil-borne pollutants, its advantages make it a valuable environmental monitoring tool, especially for studies focusing on the atmospheric deposition of pollutants.

## 4. Materials and Methods

### 4.1. Study Area

**Figure 9:**
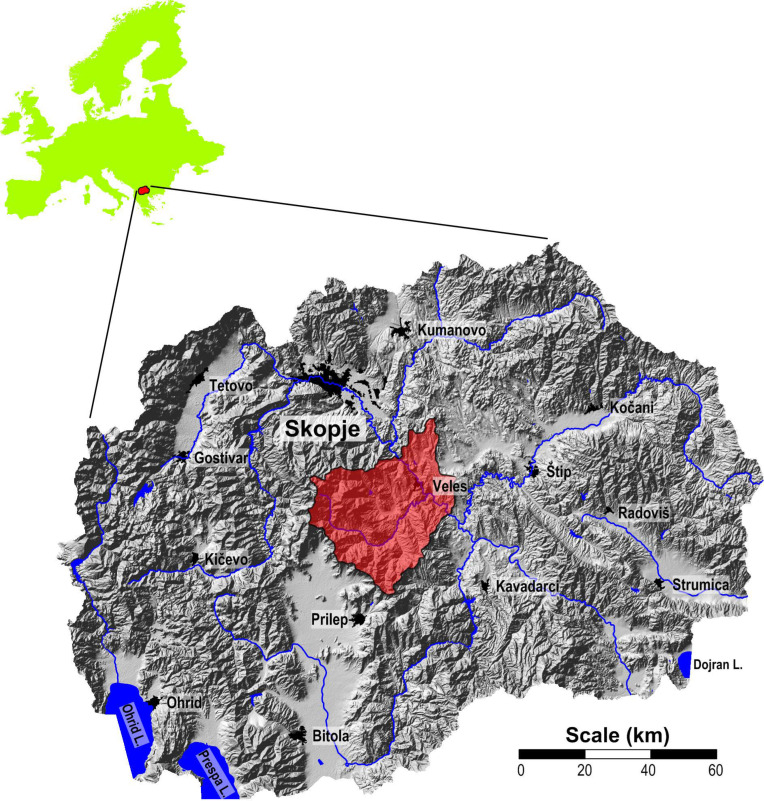
Location of the study area in North Macedonia.

The study area is located in the central part of North Macedonia (Figure 9) and includes the municipalities of Veles and Časka with an area of approximately 1200 km^2^. The region benefits from a strategic geographical location, as the Vardar River, the country’s main waterway, serves as an important transportation route. The Veles Valley is surrounded by remarkable mountain ranges: the Taorska Gorge and Mount Orešnica to the north, Mount Klepa to the south, and Mount Jakupica and Mount Babuna to the west. The Babuna River, an important tributary of the Vardar, has a significant influence on the hydrology of the region.

Veles is one of the largest cities in North Macedonia. In the 2021 census, 62,700 people lived in the region—55,000 in the municipality of Veles and 7700 in the municipality of Časka. Around 40,700 of these inhabitants live in the town of Veles itself. The town is located at an altitude of 160 to 200 m above sea level.

The climate in Veles is continental, with hot summers and cold winters. The average annual temperature is 13.4 °C, with winter temperatures ranging from −1.5 °C to 5.7 °C in January and summer temperatures ranging from 23 °C to 42.5 °C in July. The region receives an average of 469 mm of rainfall per year, which contributes to arid conditions, reduced water availability, and increased vulnerability to droughts due to the limited amount, further worsening climatic and ecological conditions [34,36].

This region, with its diverse geographical features and climatic characteristics, provides a representative setting for assessing the environmental impact of industrial and urban activities, particularly the distribution of potentially toxic elements (PTEs) in air, soil, and vegetation.

### 4.2. Geological Description

**Figure 10:**
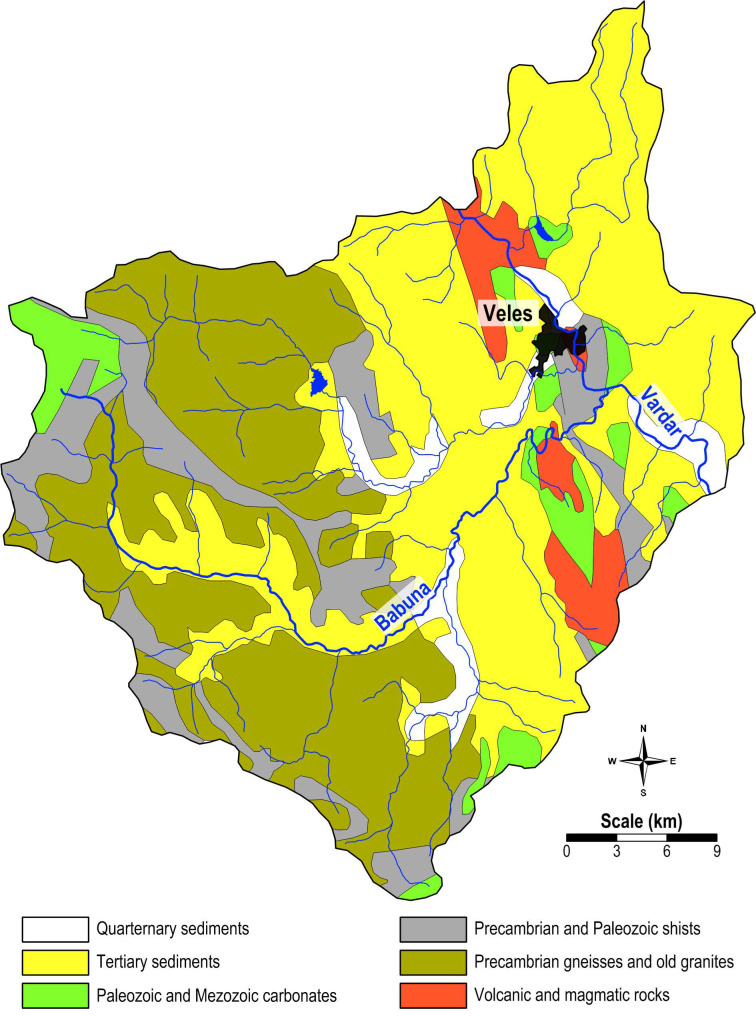
Geological map of the study area.

The geology of the study area is highly diverse. The geological map (Figure 10) shows that Tertiary sediments predominate in the eastern and central parts and in the area along the Babuna River. In the central eastern part, along the Vardar River, volcanic and igneous rocks, Paleozoic and Mesozoic carbonates, and Precambrian and Paleozoic shales are present in addition to the Tertiary sediments. The western part of the study area is dominated by Precambrian gneisses and ancient granites, with rare occurrences of Precambrian and Paleozoic schists and Paleozoic and Mesozoic carbonates. Quaternary sediments can also be found in some areas along the Vardar, Babuna, and Topolka rivers.

The lithology around the town of Veles is equally diverse. The oldest rocks, dating from the Paleozoic era, belong to an inner part of the Vardar zone. This Paleozoic series consists of green schist, feldspathic schist, biotite schist, and quartz-sericite schist interspersed with layers of marble and quartzite. The subsequent Mesozoic carbonate rocks, which also belong to the Vardar Zone, are predominantly found in the west of the study area. Pliocene sand and clay cover part of this area. The youngest sediments are alluvial deposits of the Vardar River and, to a certain extent, of the Topolka River [28,34].

### 4.3. Moss and Soil Sampling, Preparation, and Analysis

Moss sampling followed the guidelines outlined in the Monitoring Manual of the United Nations Economic Commission for Europe under the Convention on Long-range Transboundary Air Pollution (ICP Vegetation). According to these guidelines, moss samples should not be washed before analysis in order to preserve the integrity of the elements absorbed by the moss [37].

**Figure 11:**
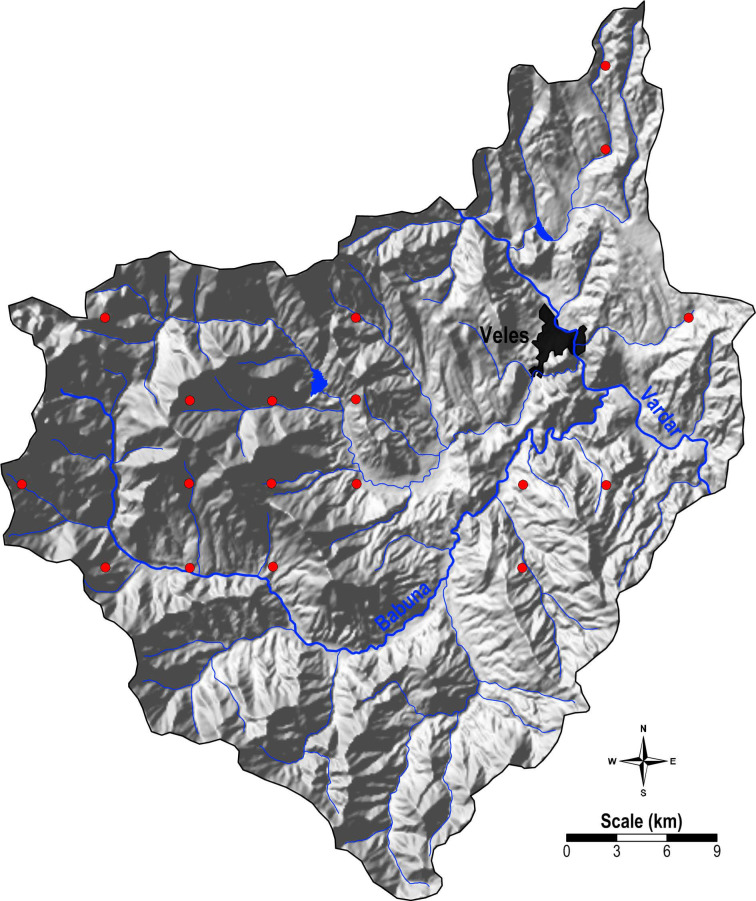
Study area with sampling locations (The red dots represent the locations where the moss and soil samples were taken).

During the summer of 2014, a total of 18 moss samples were collected from the study area, adhering to a 5 × 5 km grid pattern (Tables S1 and S2 in the Supplementary Material). The collection included 11 samples of *Camptothecium lutescens* and 7 samples of *Hypnum cupressiforme*. A map showing the locations of the moss samples can be seen in Figure 11.

The moss samples (0.5 g) were placed in Teflon digestion vials, to which 5 mL of concentrated nitric acid (HNO_3_, 69%) and 2 mL of hydrogen peroxide (H_2_O_2_, 30%, m/V) were added. Sample digestion was performed using a Mars Microwave Digestion System (Mars, CEM, USA). The digestion was carried out with the following program: step 1: temperature 180 °C, 5 min ramp time, with power of 500 W and 20 bar pressure; step 2: temperature 180 °C, 5 min holding time, with power of 500 W and 20 bar pressure. After cooling, the digested samples were quantitatively transferred into calibrated 25 mL flasks. The concentrations of 21 chemical elements (Ag, Al, As, Ba, Ca, Cd, Cr, Cu, Fe, K, Li, Mg, Mn, Mo, Na, Ni, P, Pb, Sr, V, and Zn) were determined using inductively coupled plasma–atomic emission spectrometry (ICP-AES, Varian 715-ES) under optimized operating conditions [38]. Quality control was ensured by using the standard moss reference materials M2 and M3, prepared for the European moss survey [39]. The measured concentrations were within the recommended values, with differences between measured and certified values remaining within 15%.

Soil samples were collected from the same 18 predetermined locations as the moss samples (Figure 11). At each site, topsoil (0–5 cm) and subsoil (20–30 cm) were sampled from five sub-sites within a 10 m radius [40]. The collected samples were placed in plastic bags, cleared of foreign material, air-dried at room temperature, crushed, sieved through a 2 mm mesh, and finely ground in an agate mill to achieve a particle size below 0.1 mm.

Wet acid digestion was performed using a mixture of HNO_3_, HClO_4_, HF, and HCl in accordance with ISO 14869-1:2001 [41]. The resulting solution was filtered and quantitatively transferred into a 25 mL volumetric flask, which was then brought to volume with distilled water. The concentrations of 19 elements (Al, B, Ba, Ca, Cd, Cr, Cu, Fe, K, Li, Mg, Mn, Na, Ni, P, Pb, Sr, V, and Zn) were determined using inductively coupled plasma–atomic emission spectrometry (ICP-AES, Varian 715-ES).

To ensure analytical accuracy, quality control measures included the analysis of a certified soil reference material (JSAC 0401-Japan Society for Analytical Chemistry, Tokyo, Japan) and spiked intralaboratory samples, accounting for 20% of the total samples. The recovery rates ranged from 90% to 110% for spiked samples and from 94% to 108% for the certified reference material.

### 4.4. Advantages and Limitations of Moss Biomonitoring

#### 4.4.1. Advantages of Moss Biomonitoring

Non-destructive sampling. Mosses are non-invasive and do not require the destruction of the environment, making them an ethical and sustainable choice for long-term monitoring.

Sensitive to atmospheric deposition. Mosses accumulate elements directly from the atmosphere, providing valuable information about pollutants in the air that are difficult to detect with other methods.

Cost-effective. Moss sampling is relatively inexpensive and simple compared to other biomonitoring methods, such as the use of lichens or tree rings.

Widespread distribution. Moss species are widely distributed and can be found in different ecosystems, making them accessible for large-scale monitoring projects.

#### 4.4.2. Limitations in Moss Biomonitoring

Limited to atmospheric pollution: Mosses primarily reflect atmospheric deposition and are limited in their ability to detect soil pollution. This means that they may not capture all pollutants, especially those that are mainly soil-bound or located in deeper soil layers.

Species variability: Different moss species can accumulate elements at different rates, making comparisons between studies or regions difficult unless the same species is used consistently.

Lack of a root system: Mosses do not have roots, which means that they cannot reflect soil-borne pollutants as accurately as plants with a deeper root system.

Environmental factors: Factors such as temperature, humidity, and air circulation can affect the accumulation of elements in mosses, leading to potential variability in results.

#### 4.4.3. Comparison with Other Biomonitoring Methods

Soil sampling: Soil is a more stable medium compared to moss and captures both atmospheric and geochemical sources of pollution. However, soil sampling is more invasive and expensive compared to moss, and the analysis of soil requires more complex sample preparation.

Lichen sampling: Lichens are also good biomonitors of air pollution, but their growth is often slower than that of mosses, and they are more sensitive to environmental changes such as humidity and temperature. In addition, lichen species are less widely distributed compared to mosses, which limits their use in large-scale studies.

Plant-based biomonitoring: Other plant species, especially those with deep root systems, are more suitable for assessing soil contamination. However, their use is often more complex and expensive compared to mosses, as they require detailed monitoring of growth and sampling over long periods of time.

### 4.5. Data Processing and Statistical Analysis

The interpretation and visualization of the geostatistical data were performed with the following software: Statistica Ver. 14 (Stat Soft, Inc.), Autodesk MAP 3D (AutoCAD Map 3D), ArcIn-fo (ArcGIS Pro 3.0) and Surfer Ver. 25 (Golden Software, Inc.). Both parametric and non-parametric statistical methods were applied [42,43], and normality tests were performed to evaluate the data distributions.

Due to the different value ranges and units of the variables, standardization was performed to ensure equal weighting during the analysis and to mitigate the effects of similarity between items. The data were also transformed to reduce the differences between the extreme values. In particular, a Box-Cox transformation, commonly used in environmental and geosciences [44,45,46,47], was applied based on the results and previous experience.

Multivariate cluster analysis and R-mode factor analysis (FA) were used to investigate the relationships between the chemical elements [33,48]. FA was performed with standardized variables (mean = 0, standard deviation = 1) using the varimax method for orthogonal rotation. The spatial distribution of factor values and trace elements was mapped using the universal kriging method with linear variogram interpolation. The base grid cell size for interpolation was set to 25 × 25 m. Seven percentile-based classes (0–10, 10–25, 25–40, 40–60, 60–75, 75–90, and 90–100) were defined as class boundaries for the interpolated value distributions [49].

## 5. Prospects

**Long-term monitoring**: Ongoing biomonitoring of moss can track pollution trends and support modeling of future pollution levels.

**Expanded spatial coverage**: Extending the monitoring network to more locations can improve understanding of regional pollution patterns and identify hotspots.

**Policy integration**: The data can provide targeted strategies to combat pollution and improve environmental policy.

**Comparative studies**: Comparing moss with other biomonitors could provide a more comprehensive assessment of pollutant distribution.

**Raising public awareness**: The findings can help raise awareness about air quality and advocate for stricter environmental regulations.

These prospects aim to guide future research, policy development, and environmental management in North Macedonia.

## Figures and Tables

**Table 1:** Descriptive statistics for the content of analyzed elements in moss samples (*n* = 18).

Elements	Unit	X	Md	Min	Max	P_10_	P_90_	P_25_	P_75_	S	S_x_	CV	A	E
Ag	µg/kg	99	73	29	430	33	170	53	110	91	21.5	92.1	3.19	11.68
Al	%	0.19	0.16	0.069	0.49	0.088	0.37	0.13	0.23	0.11	0.025	56.4	1.56	2.32
As	mg/kg	0.88	0.66	0.25	2.5	0.25	2.2	0.25	1.3	0.67	0.16	76.6	1.35	1.39
Ba	mg/kg	22	21	7.7	48	11	41	12	25	11	2.6	49.7	0.99	0.69
Ca	%	0.49	0.48	0.31	0.64	0.38	0.62	0.44	0.54	0.087	0.021	17.9	−0.05	−0.19
Cd	mg/kg	0.18	0.20	0.047	0.35	0.05	0.28	0.10	0.25	0.089	0.021	48.3	−0.06	−0.84
Cr	mg/kg	3.7	2.7	1.7	12	1.9	7.1	2.2	4.2	2.6	0.60	69.3	2.12	4.76
Cu	mg/kg	6.2	4.9	3.2	13	3.7	11	4.0	7.9	2.9	0.68	46.8	1.17	0.57
Fe	%	0.15	0.12	0.062	0.34	0.080	0.28	0.11	0.17	0.07	0.02	48.9	1.51	1.99
K	%	0.28	0.26	0.14	0.49	0.19	0.35	0.24	0.31	0.07	0.02	26.2	1.14	3.53
Li	mg/kg	1.5	1.3	0.53	3.7	0.75	3.2	0.96	1.7	0.84	0.20	56.1	1.53	2.09
Mg	%	0.12	0.12	0.071	0.17	0.085	0.16	0.10	0.14	0.03	0.01	22.1	−0.18	−0.47
Mn	mg/kg	62	60	23	110	33	94	43	76	24	5.5	38.1	0.46	−0.25
Mo	µg/kg	130	100	50	350	50	300	50	210	96	22.6	74.2	1.10	0.34
Na	mg/kg	99	90	71	230	73	130	85	96	35	8.2	35.0	3.20	11.50
Ni	mg/kg	5.7	4.8	3.1	13	3.2	9.5	3.7	6.4	2.6	0.62	45.8	1.51	2.35
P	mg/kg	570	550	310	900	350	830	490	670	150	36.4	27.0	0.35	−0.06
Pb	mg/kg	2.1	2.1	0.93	3.3	1.2	2.9	1.7	2.6	0.65	0.15	30.8	0.10	−0.62
Sr	mg/kg	16	16	8.3	25	8.4	22	12	19	4.7	1.10	29.8	0.13	−0.69
V	mg/kg	2.8	2.2	1.1	7.2	1.4	4.7	1.7	3.9	1.6	0.37	56.5	1.61	2.71
Zn	mg/kg	27	27	18	39	20	32	23	30	5.1	1.19	19.0	0.38	0.60

X—mean, Md—median, Min—minimum, Max—maximum, P_10_—10 percentile, P_25_—25 percentile, P_75_—75 percentile, P_90_—90 percentile, S—standard deviation, S_x_—standard deviation (standard error), CV—coefficient of variation, A—skewness, E—kurtosis.

**Table 2:** Descriptive statistics for the content of analyzed elements in topsoil samples (*n* = 18).

Element	Unit	X	Md	Min	Max	P_10_	P_90_	P_25_	P_75_	S	Sx	CV	A	E
Ag	mg/kg	1.25	0.94	0.34	2.87	0.46	2.65	0.56	1.86	0.89	0.21	71.5	0.89	−0.82
Al	%	19.3	18.6	7.87	42.5	9.20	31.2	11.4	24.0	9.77	2.30	50.4	0.82	0.17
Ba	mg/kg	690	363	147	3475	226	1618	246	481	946	223	137	2.55	5.58
Ca	%	22.1	9.09	3.37	112	4.46	64.2	5.42	16.3	30.0	7.07	135	2.17	4.26
Cd	mg/kg	2.25	2.25	2.03	2.48	2.07	2.44	2.13	2.37	0.20	0.10	8.7	0.11	−1.76
Cr	mg/kg	105	86.3	36.6	353	45.7	162	56.3	124	76.7	18.0	72.8	2.28	6.18
Cu	mg/kg	12.2	12.1	3.20	21.8	4.30	19.6	8.45	18.1	5.89	1.39	48.1	0.05	−1.19
Fe	%	20.5	18.2	9.54	34.5	12.3	31.0	15.1	26.6	7.44	1.75	36.3	0.48	−0.95
K	%	15.0	15.0	8.58	23.4	10.0	20.4	12.8	17.2	4.04	0.95	26.8	0.26	−0.13
Li	mg/kg	13.4	14.2	6.23	25.3	8.09	18.4	9.21	15.9	4.84	1.14	35.9	0.65	0.57
Mg	%	4.58	4.14	1.51	9.44	2.21	8.09	2.53	6.42	2.49	0.59	54.4	0.69	−0.63
Mn	mg/kg	489	451	200	1455	221	706	276	560	298	70.2	61.0	2.12	6.07
Mo	mg/kg	8.54	6.44	5.99	13.2	5.99	12.3	6.00	11.0	3.38	1.51	39.6	0.83	−1.96
Na	%	12.5	13.3	3.12	26.4	3.87	17.5	8.59	16.2	5.98	1.41	47.7	0.23	0.40
Ni	mg/kg	54.9	23.6	6.81	233	10.0	133	17.4	75.9	62.4	14.7	113	1.76	2.82
P	mg/kg	262	264	106	476	116	381	189	302	102	24.1	39.2	0.30	0.05
Pb	mg/kg	26.4	25.6	16.3	51.7	17.2	33.3	19.9	29.9	8.76	2.07	33.1	1.49	3.09
Sr	mg/kg	67.6	62.2	25.1	151	33.5	103	43.5	76.0	35.2	8.31	52.2	1.43	2.05
V	mg/kg	49.5	44.5	23.7	90.3	28.7	70.8	37.8	63.8	19.3	4.55	38.9	0.74	−0.16
Zn	mg/kg	86.0	82.2	61.9	128	64.9	107	68.6	99	19.4	4.58	22.6	0.63	−0.40

X—arithmetic mean, Md—median, Min—minimum, Max—maximum, P_10_—10 percentiles, P_90_—90 percentiles, P_25_—25 percentiles, P_75_—75 percentiles, S—standard deviation, S_x_—standard deviation (standard error), CV—coefficient of variation, A—skewness, E—kurtosis.

**Table 3:** Comparison of the results of the present study (Veles region) for the moss samples with those from the whole area of North Macedonia collected in 2015 (in mg/kg).

Element	Veles Region (Present Work); *n* = 18		North Macedonia, 2015 [27]; *n* = 72	
	Median	Range	Median	Range
Ag	0.099	0.029–0.43	-	-
Al	1900	690–4900	2100	750–7400
As	0.88	0.25–2.5	0.48	0.13–1.4
Ba	22	7.7–48	42	9.7–180
Ca	4900	3100–6400	6900	3500–13,000
Cd	0.18	0.047–0.35	0.23	0.018–0.88
Cr	3.7	1.7–12	5.7	1.8–31
Cu	6.2	3.2–4.9	4.6	3.0–8.3
Fe	1500	620–3400	1700	510–4600
K	2800	1400–4900	6000	3100–14,000
Li	1.5	0.53–3.7	0.79	0.32–3.5
Mg	1200	710–1700	1900	1200–1200
Mn	62	23–110	160	33–510
Na	99	71–230	190	140–380
Ni	5.7	3.1–13	3.5	0.68–63
P	570	310–900	1100	420–2000
Pb	2.1	0.93–3.3	4.9	2.2–14
Sr	16	8.3–2.2	25	6.5–220
V	2.8	1.1–7.2	3.3	0.47–11
Zn	27	18–39	30	12–66

**Table 4:** Pearson correlation coefficient between the element contents in mosses from Veles region (group of 16 elements determined by ICP-AES). Values in the range 0.45–0.7 (good association) are underlined and in the range 0.7–1.0 (strong association) are in bold; Box-Cox-transformed values were used.

Element	Ag	Al	As	Ba	Ca	Cd	Cr	Cu	Fe	K	Li	Mg	Mn	Mo	Na	Ni	P	Pb	Sr	V	Zn
Ag	1.00																				
Al	0.36	1.00																			
As	0.45	0.36	1.00																		
Ba	−0.01	**0.79**	0.38	1.00																	
Ca	−0.05	0.28	−0.13	0.37	1.00																
Cd	0.60	0.34	0.00	0.02	0.03	1.00															
Cr	−0.02	0.59	−0.18	0.36	0.35	0.16	1.00														
Cu	−0.02	0.38	0.39	0.45	0.04	−0.23	0.45	1.00													
Fe	0.34	**0.99**	0.33	**0.78**	0.26	0.39	0.61	0.34	1.00												
K	−0.41	0.09	0.10	0.35	0.29	−0.67	0.08	0.47	0.04	1.00											
Li	0.38	**0.95**	0.38	**0.73**	0.26	0.37	0.45	0.28	**0.95**	0.07	1.00										
Mg	−0.35	0.34	0.13	0.56	0.43	−0.43	0.45	**0.72**	0.30	0.66	0.32	1.00									
Mn	0.25	**0.79**	0.28	0.55	0.07	0.31	0.46	0.39	**0.80**	0.25	**0.83**	0.26	1.00								
Mo	−0.14	−0.02	−0.31	−0.19	0.23	−0.20	0.19	−0.11	−0.05	0.38	−0.10	−0.02	0.11	1.00							
Na	−0.05	0.59	0.34	0.69	0.07	−0.04	0.29	0.31	0.59	0.11	0.41	0.16	0.41	−0.10	1.00						
Ni	0.06	0.48	−0.21	0.19	0.29	0.28	**0.95**	0.41	0.50	−0.03	0.35	0.36	0.38	0.18	0.13	1.00					
P	−0.31	0.20	0.17	0.49	0.21	−0.56	0.20	0.65	0.16	**0.89**	0.11	0.67	0.35	0.21	0.35	0.06	1.00				
Pb	0.26	0.60	−0.05	0.41	0.14	0.54	0.68	0.37	0.64	−0.09	0.54	0.23	0.61	−0.02	0.28	**0.74**	0.05	1.00			
Sr	0.11	0.42	0.25	0.48	0.64	−0.12	−0.05	0.08	0.36	0.52	0.46	0.41	0.29	0.35	0.17	−0.12	0.36	−0.03	1.00		
V	0.34	**0.95**	0.27	**0.75**	0.22	0.38	**0.71**	0.41	**0.96**	0.01	**0.84**	0.29	**0.73**	−0.06	0.65	0.62	0.19	**0.72**	0.22	1.00	
Zn	0.29	0.44	0.32	0.51	0.13	0.32	0.29	0.51	0.46	0.24	0.46	0.37	0.53	−0.35	0.17	0.32	0.38	0.67	0.09	0.50	1.00

**Table 5:** Paired samples to determine the statistically significant difference between the concentrations of each element in soil and moss samples.

Soil	Moss	*t*	*df*	*p*
Ag_soil	Ag_moss	5.35	17.00	<0.001
Al_soil	Al_moss	7.30	17.00	<0.001
As_soil	As_moss	49.75	12.00	<0.001
Ba_soil	Ba_moss	3.00	17.00	0.008
Ca_soil	Ca_moss	2.44	17.00	0.026
Cd_soil	Cd_moss	18.04	3.00	<0.001
Co_soil	Co_moss	NaN ^a^		
Cr_soil	Cr_moss	5.62	17.00	<0.001
Cu_soil	Cu_moss	4.14	17.00	<0.001
Fe_soil	Fe_moss	10.41	17.00	<0.001
K_soil	K_moss	13.30	17.00	<0.001
Li_soil	Li_moss	9.94	17.00	<0.001
Mg_soil	Mg_moss	5.94	17.00	<0.001
Mn_soil	Mn_moss	5.90	17.00	<0.001
Mo_soil	Mo_moss	3.49	2.00	0.073
Na_soil	Na_moss	8.82	17.00	<0.001
Ni_soil	Ni_moss	3.36	17.00	0.004
P_soil	P_moss	−15.61	17.00	<0.001
Pb_soil	Pb_moss	11.59	17.00	<0.001
Sr_soil	Sr_moss	5.99	17.00	<0.001
V_soil	V_moss	10.06	17.00	<0.001
Zn_soil	Zn_moss	11.96	17.00	<0.001

H_a_: μ(Measure 1) − μ(Measure 2) ≠ 0; *t*—Student’s *t*-test; *df*—Degree of freedom; *p*—Probability; ^a^ NaN—Not a Number.

## Data Availability

The datasets used in this study are available from the corresponding author upon reasonable request.

## Supplementary Materials

The following supporting information can be downloaded at: https://www.mdpi.com/article/10.3390/plants14050783/s1.
