# Supplementary material for: Assessing the Distribution of Potentially Toxic Elements in Bryophytes in Relation to Surface Soil Contamination in the Veles Region, North Macedonia
Source: Plants (Basel). 2025 Mar 4;14(5):783. doi: 10.3390/plants14050783 (PMC11901712; doi:10.3390/plants14050783)
Supplement: Supplementary file 1 [file plants-14-00783-s001.zip › plants-3489546-supplementary.pdf]

Table SM1. Data on sampling locations and moss species

	Veles-moss	N	E	Elevation, m	Location	Species
**1**	MK-493	41° 35′ 40″	21° 26′ 41″	764	w. Bogomila	*Campthotecium lutescens*
**2**	MK-494	41° 35′ 38″	21° 30′ 20″	470	w. Bogomila	*Campthotecium lutescens*
**3**	MK-495	41° 35′ 39″	21° 33′ 55″	479	w. Sogle	*Campthotecium lutescens*
**4**	MK-498	41° 35′ 33″	21° 44′ 40″	471	w. Dolno Vranovci	*Campthotecium lutescens*
**5**	MK-533	41° 38′ 22″	21° 23′ 06″	1461	w. Orese	*Hypnum cupressiforme*
**6**	MK-535	41° 38′ 22″	21° 30′ 19″	1046	w. Cresnevo	*Campthotecium lutescens*
**7**	MK-536	41° 38′ 21″	21° 33′ 52″	911	w. Melnica	*Campthotecium lutescens*
**8**	MK-537	41° 38′ 19″	21° 37′ 33″	319	w. Elovec	*Hypnum cupressiforme*
**9**	MK-539	41° 38′ 15″	21° 44′ 44″	320	w. Vitanci	*Campthotecium lutescens*
**10**	MK-540	41° 38′ 13″	21° 48′ 19″	438	w. Crkvino	*Campthotecium lutescens*
**11**	MK-576	41° 41′ 03″	21° 30′ 22″	1578	w. Gorno Vranovci	*Hypnum cupressiforme*
**12**	MK-577	41° 41′ 01″	21° 33′ 56″	665	w. Gorno Vranovci	*Hypnum cupressiforme*
**13**	MK-578	41° 41′ 03″	21° 37′ 32″	484	w. Lisice	*Hypnum cupressiforme*
**14**	MK-616	41° 43′ 45″	21° 26′ 43″	1839	Begovo Pole	*Campthotecium lutescens*
**15**	MK-619	41° 43′ 42″	21° 37′ 34″	570	w. Rlevci	*Hypnum cupressiforme*
**16**	MK-623	41° 43′ 36″	21° 51′ 57″	343	Veles	*Hypnum cupressiforme*
**17**	MK-705	41° 49′ 05″	21° 48′ 26″	376	w. Lugunci	*Campthotecium lutescens*
**18**	MK-745	41° 51′ 47″	21° 48′ 28″	530	w. Ivankovci	*Campthotecium lutescens*

Table SM2. Data on soil sampling locations

	Veles-soil	N	E	Elevation, m	Location
1	MK-493T	41° 35′ 40"	21° 26′ 41′′	764	w. Bogomila
2	MK-494T	41° 35′ 38″	21° 30′ 20″	470	w. Bogomila
3	MK-495T	41° 35′ 39″	21° 33′ 55″	479	w. Sogle
4	MK-498T	41° 35′ 33″	21° 44′ 40″	471	w. Dolno Vranovci
5	MK-533T	41° 38′ 22″	21° 23′ 06″	1461	w. Orese
6	MK-535T	41° 38′ 22″	21° 30′ 19″	1046	w. Cresnevo
7	MK-536T	41° 38′ 21″	21° 33′ 52″	911	w. Melnica
8	MK-537T	41° 38′ 19″	21° 37′ 33″	319	w. Elovec
9	MK-539T	41° 38′ 15″	21° 44′ 44″	320	w. Vitanci
10	MK-540T	41° 38′ 13″	21° 48′ 19″	438	w. Crkvino
11	MK-576T	41° 41′ 03″	21° 30′ 22″	1578	w. Gorno Vranovci
12	MK-577T	41° 41′ 01″	21° 33′ 56″	665	w. Gorno Vranovci
13	MK-578T	41° 41′ 03″	21° 37′ 32″	484	w. Lisice
14	MK-616T	41° 43′ 45″	21° 26′ 43″	1839	Begovo Pole
15	MK-619T	41° 43′ 42″	21° 37′ 34″	570	w. Rlevci
16	MK-623T	41° 43′ 36″	21° 51′ 57″	343	Veles
17	MK-705T	41° 49′ 05″	21° 48′ 26″	376	w. Lugunci
18	MK-745T	41° 51′ 47″	21° 48′ 28″	530	w. Ivankovci
